# Changes in Cross-Sectional Area of the Median Nerve and Body Composition Parameters after Treatment of Acromegaly: 1 year Follow-Up

**DOI:** 10.1155/2022/8766046

**Published:** 2022-10-13

**Authors:** Ivana Ságová, Daniela Kantárová, Marián Mokáň, Peter Vaňuga

**Affiliations:** ^1^Department of Endocrinology, National Institute of Endocrinology and Diabetology, Ľubochňa, Slovakia; ^2^Comenius University Jessenius Faculty of Medicine, 1st Department of Internal Medicine, University Hospital Martin, Martin, Slovakia

## Abstract

**Objective:**

Median neuropathy is a common manifestation of acromegaly, although its pathology is uncertain. Changes in levels of growth hormone (GH) and insulin-like growth factor I (IGF-I) and body composition are potential parameters in pathology of median neuropathy in acromegaly. We aimed to assess changes in the cross-sectional area (CSA) of the median nerve and body composition in newly diagnosed acromegalic patients 1 year after treatment and to determine their mutual relationships.

**Design:**

This prospective study included 30 patients with newly diagnosed acromegaly and 30 healthy controls matched for age, gender, and body mass index. Physical and laboratory examinations, dual-energy X-ray absorptiometry (DXA), and ultrasound evaluations were performed at baseline and 1 year after initial treatment.

**Results:**

The CSA of the median nerve was increased in acromegalic patients compared with controls (13.1 mm^2^ [12.2–14.9] vs 7.5 mm^2^ [6.4–8.4], *P* < 0.001). One year after treatment of acromegaly, GH and IGF-I levels decreased significantly. The median nerve CSA was significantly reduced after treatment (11.6 mm^2^ [10.2–13.1], *P* < 0.001). Reduction of IGF-I levels correlated with a decrease in lean mass and increase in fat mass. The median nerve CSA positively correlated with IGF-I levels (*R* = 0.492, *P*=0.006) and lean mass (*R* = 0.419, *P*=0.021) in acromegalic patients before treatment.

**Conclusion:**

This study demonstrates a reduction in the median nerve CSA 1 year after treatment of acromegaly. These changes are closely associated with a reduction in IGF- I levels and in lean body mass. The enlargement of the median nerve in acromegaly can be reversed with adequate treatment.

## 1. Introduction

Acromegaly is characterized by overproduction of growth hormone (GH) and insulin-like growth factor I (IGF-I). Carpal tunnel syndrome (CTS) is a common manifestation of acromegaly, which occurs in approximately 20% to 64% of acromegalic patients at the time of diagnosis [1, 2]. The pathology remains unclear. CTS in acromegaly has various proposed pathogenic mechanisms, including an increased amount of connective tissue in the carpal tunnel, demyelination of Schwann cells, bone or synovial overgrowth of carpal bones, or an increased amount of extracellular fluid in the tunnel [1]. IGF-I interferes with CTS pathogenesis by several mechanisms.

IGF-I is a neuropathic factor, promoting nerve elongation and branching, and a myogenic factor, promoting cell proliferation, differentiation, and muscle hypertrophy [3]. GH/IGF-1 also increases activity of the epithelial sodium channel, which could contribute to volume expansion and soft tissue manifestation [4]. Several studies have found that the predominant CTS pathology in acromegaly consisted of increased edema of the median nerve in the carpal tunnel rather than extrinsic compression due to increased volume of the carpal tunnel contents [3, 5]. Nerve edema improves when GH and IGF-I levels decrease, suggesting that hormonal control is key in improving neurological status [6]. CTS diagnosis is typically based on patient history, physical examination, and electrodiagnosis [7].

High-resolution ultrasonography (US) is a highly valuable tool for CTS diagnosis [7, 8]. The most widely used ultrasonography method in CTS diagnosis is measuring the cross-sectional area (CSA) of the median nerve at the level of the inlet of the carpal tunnel (pisiform bone level). US sensitivity has been reported to be as high as 97% [9]. Normal ranges for the median nerve area vary among the reports, ranging from 7.2 to 9.8 mm^2^ [10–12]. The most commonly used US parameter to diagnose CTS is the median nerve CSA at an inlet of the carpal tunnel ≥10 mm^2^ [9, 13].

Acromegaly is also associated with changes in body composition. Overproduction of GH/IGF-I leads to protein synthesis stimulation and increased lipolysis in adipose tissue. Active acromegaly is associated with decreased total body fat and increased lean body mass and total body water [14, 15]. These changes are associated with severity of the disease and GH/IGF-I levels, and are normalized after successful treatment [16]. Changes in body composition caused by IGF-I overproduction could be a potential factor in carpal tunnel pathogenesis in acromegaly.

The aim of our study was to investigate the median nerve CSA (by ultrasound) and body composition parameters (by DXA) in newly diagnosed acromegalic patients compared with healthy controls at the baseline and to determine changes in these parameters 1 year after treatment of acromegaly. We wanted to analyse the mutual relationships between body composition parameters and the median nerve CSA in order to identify their potential as risk factors for CTS in acromegaly.

## 2. Materials and Methods

This prospective observational study was performed at the National Institute of Endocrinology and Diabetology in Ľubochňa, between June 2016 and January 2022. The study protocol was approved by the regional medical ethics committee. Every study subject voluntarily signed an informed consent form for study participation.

### 2.1. Patients and Controls

We prospectively examined 30 newly diagnosed acromegalic patients (18 females and 12 males) and a control group of 30 healthy volunteers (18 females and 12 males) matched for sex, age, and body mass index (BMI).

### 2.2. Acromegalic Patients

- The inclusion criterion for newly diagnosed acromegalic patients was presence of acromegaly. The diagnosis was based on the Endocrine Society Clinical Practice Guideline: GH levels after the oral glucose tolerance test (oGTT) > 1 *µ*g/l and IGF-I levels above the normal range for age and sex [17].
- No history of acromegaly treatment.

### 2.3. Healthy Age-Matched, Sex-Matched, and BMI-Matched Control Subjects

- Subjects without Presence of Acromegaly (Assessed by Normal IGF-I Values)

The exclusion criteria for all subjects were as follows: (a) presence of diabetes mellitus and hypothyroidism, (b) a history of wrist trauma, (c) a history of familial neuropathy, (d) a history of alcohol abuse, (e) neuropathies due to chronic renal failure, liver disease, pregnancy, and cervical neuropathy, (f) presence of vitamin B12 deficiency, and (g) a history of polyneuropathy and paraneoplastic inflammation.

In acromegalic patients, the first (baseline) clinical history, physical examination, laboratory examinations, DXA, and US were performed at the time of diagnosis of acromegaly before the treatment. The second complete examinations with control DXA and US were made 1 year after the start of the treatment (1 year after transsphenoidal surgery and/or 1 year after somatostatin analogue therapy). Transsphenoidal surgery was performed on 22 acromegalic patients; eight of them received primary medical treatment with somatostatin analogue before the surgery. After the surgery, 14 patients were cured, and 8 patients underwent postsurgery somatostatin analogue therapy. During the first 3 postoperative months, we identified 2 patients with hypothyroidism, 3 patients with transient adrenal insufficiency (2 weeks after surgery), and 2 patients with hypogonadism. To maintain normal pituitary function, hormone substitution treatment was applied. The eight patients who did not undergo surgery were primarily treated with somatostatin analogue, and this treatment continued. In 2 patients, GH receptor antagonist cotreatment was added. None of the patients underwent further surgery during the study duration or underwent radiotherapy. One year after the start of the treatment, the disease was under control in 19 out of the 30 acromegalic patients, using the Endocrine Practice Guidelines [17].

### 2.4. Clinical Examination

In all study subjects, anthropometric measurements including weight (kg) and height (cm) were performed. Body weight and height were measured in light indoor clothing without shoes. Standing height was measured using a wall-mounted Harpenden stadiometer (Holt AIM Ltd., UK). Body weight was measured using a calibrated electronic scale (SECA 877, Germany). BMI was calculated as weight in kilograms divided by the square of height in meters. Information about the presence of pain, weakness, and numbness symptoms of paraesthesia was collected.

### 2.5. Laboratory Assays

In all study subjects, we measured serum levels of GH, IGF-I, pituitary hormones, blood count differential, creatinine concentration, CRP level, liver enzymes, serum glucose, glycated haemoglobin, TSH, free T4, and vitamin B12. Venous blood samples were taken after overnight fasting (minimum 8 h of fasting) between 07 : 00 and 08 : 00 am. IGF-I and GH levels were assessed with the ECLIA chemiluminescent immunometric assay (Immulite 2000 assay, Siemens Healthcare Diagnostics Products Ltd., United Kingdom). Intra-assay variability (CV) for IGF-I is between 3.0 and 7.6%, and for GH, it is between 6.5 and 6.6%. The normal level for serum GH was 5 ng/ml. The normal range of IGF-1 was adjusted for sex and age.

### 2.6. Ultrasound

Ultrasound of the carpal tunnel was performed using a 12–5 MHz linear array transducer (Hitachi-Hi Preirus, ultrasound machine, Tokyo, Japan). All patients underwent ultrasound of the carpal tunnel before the treatment of acromegaly and 1 year after. Ultrasound was performed with the patient seated opposite the sonographer, with the wrists placed in a horizontal supine position on the examination table, and with fingers semiextended. The CSA of the median nerve was measured at the carpal tunnel inlet, between the pisiform bone and the scaphoid tubercle, where the distal volar crease is an external pisiform landmark. The median nerve was evaluated in the transverse plane. On transverse sonograms, the median nerve appears as an elliptic or oval outline hypoechoic reticular area with hyperechoic lines corresponding to the epineurium [18]. A longitudinal view was used to confirm correct identification of the median nerve. The median nerve CSA was calculated using the direct tracing method by outlining the perimeter just inside the hyperechoic epineurium, which marked the border of the median nerve, and the area within was measured as a cross-sectional area [19]. Each measurement was performed 5 times by the same examiner, and the mean value was used for analyses.

### 2.7. Dual Energy X-Ray Absorptiometry (DXA)

Fat body mass and lean body mass were measured by dual-energy X-ray absorptiometry (DXA) (Hologic Horizon A, Bedford, MA), using whole-body software version 13.6. The coefficient of variation was 0.78% for fat mass and 0.52% for lean mass. All patients underwent DXA before the treatment of acromegaly and 1 year after.

### 2.8. Statistical Analyses

All statistical analyses were performed using IBM SPSS version 25 (IBM SPSS Statistics, IBM Corporation, IL, USA). The data are presented as median (interquartile range). To compare patients and control, the Mann Whitney *U* test was used. The Wilcoxon signed ranks test was used for a paired comparison for the single time-point repeated measures of each patient at the beginning of the study and after 1 year (no patients dropped out). Correlation analyses were performed using the Spearman correlation coefficient. The criteria for statistical significance in all statistical tests were *p* ≤ 0.05.

## 3. Results

### 3.1. Baseline Characteristics of Subjects at the Time of Diagnosis

In total, 30 newly diagnosed acromegalic patients (18 females and 12 males) were included in the study. The age-matched, sex-matched, and BMI-matched controls consisted of 30 healthy subjects (18 females and 12 males). The median age of acromegalic patients was 52 years (43–59). The median age in controls was 53 years (42–61). Baseline characteristics of subjects are summarised in Table 1.

In acromegaly patients at diagnosis, the median IGF-I level was 526 [372–686] ng/ml with GH of 6.50 [3.03–10.79] ng/ml. Serum levels of GH and IGF-I were significantly higher in acromegalic patients than those in controls (Table 1). The cross-sectional area of the median nerve was increased in acromegalic patients compared with that of the controls (13.1 mm^2^ [12.2–14.9] vs 7.5 mm^2^ [6.4–8.4], *P* < 0.001) (Table 1). There was no statistically significant difference in BMI and fat mass between acromegalic patients and the control group, but lean mass was higher in acromegalic patients than that in the controls (60.4 kg [47.1–74.9] vs 49.1 kg [42.8–66.1], *P*=0.033) (Table 1). At the time of baseline evaluation, 7 acromegalic patients had clinical symptoms of CTS (e.g. pain, weakness, paraesthesia, and numbness).

### 3.2. Association between Activity of Acromegaly, Body Composition Parameters, and CSA of the Median Nerve in Acromegalic Patients at Baseline

**Figure 1:**
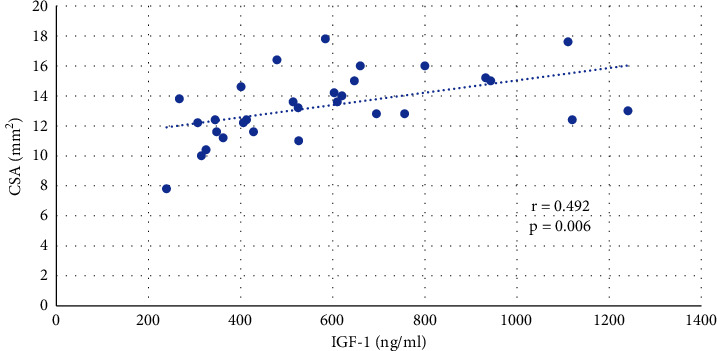
Correlation between IGF-1 and CSA of the median nerve in acromegalic patients.

**Figure 2:**
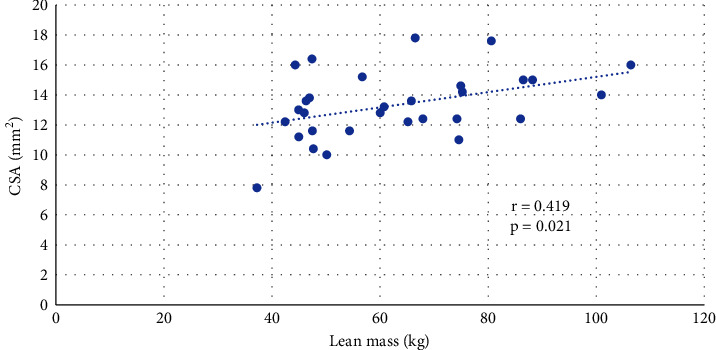
Correlation between lean body mass and the CSA of the median nerve in acromegalic patients.

A positive correlation was found between the serum levels of IGF-I and CSA of the median nerve in acromegalic patients (*R* = 0.492, *P*=0.006) (Figure 1). The relationship between CSA of the median nerve and serum levels of GH was not confirmed. No correlation was observed between BMI, fat mass, and CSA of the median nerve, but lean mass positively correlated with CSA in acromegalic patients (0.419, *P*=0.021) (Figure 2). IGF-I positively correlated with lean mass (*R* = 0.392, *P*=0.032). No correlation was observed between serum levels of IGF-I and BMI or between serum levels of IGF-I and fat mass.

### 3.3. Follow-up (1 Year after the Treatment of Acromegaly)

Biochemical parameters, CSA of the median nerve, and body composition parameters of 30 patients with acromegaly at the time of diagnosis and after the median of 12 months after initial treatment are shown in Table 2. In patients, the follow-up GH and IGF-I levels were significantly reduced (both *P* < 0.001), and there was a significant decrease in the CSA of the median nerve after treatment (13.1 mm^2^ [12.2–14.9] vs 11.6 mm^2^ [10.2–13.1], *P* < 0.001). There was no difference in BMI, but fat mass increased and lean mass decreased 1 year after the treatment (Table 2). Clinical symptoms of CTS in 5 patients were resolved after the treatment. In 2 patients, the symptoms of CTS persisted. Their median CSA was greater than 11 mm^2^ despite its reduction.

## 4. Discussion

This prospective study analysed changes in the CSA of the median nerve and body composition parameters in newly diagnosed acromegalic patients 1 year after the initial treatment of acromegaly and determined the mutual relationships. The strengths of our study are the prospective design and implementation methods. The limitation is the relatively small sample size due to the low disease incidence and the gender differences in the number of participating patients.

In this study, all acromegalic patients had significantly higher GH and IGF-I levels than controls. We found the enlargement of the median nerve in acromegalic patients compared to that of healthy controls. The increase in the median nerve CSA in acromegaly is consistent with previous research [7, 19, 20]. In acromegalic patients, the median nerve CSA significantly correlated with IGF-I levels. One year after the initial acromegaly treatment, the CSA of the median nerve significantly decreased. A correlation between the decreasing size of the median nerve and decreasing levels of IGF-I after the acromegaly treatment confirms the meaningful impact of IGF-I in CTS pathogenesis in acromegaly. Our findings are in agreement with those of other studies that describe the biochemical activity of acromegaly as a risk factor for CTS [21–23]. In our previous cross-sectional study on 107 acromegalic patients, we also found enlargement of the median nerve, which was proportional to the degree of IGF-1 levels but not dependent on the disease duration [19]. On the other hand, some studies did not confirm this association [1, 5]. The exact role of IGF-1 in pathology of CTS in acromegaly remains uncertain.

GH/IGF-I hypersecretion is associated with volume expansion, which reflects sodium-retaining properties of GH/IGF-I, stimulation of renin-angiotensin-aldosterone, atrial natriuretic factor, prostaglandins, and nitric oxide production [24]. The major predominant pathophysiological mechanisms of the median nerve neuropathy in acromegaly seem to be increased edema of the median nerve rather than extrinsic compression due to increased volume of the carpal tunnel contents [1, 3, 19, 21]. Based on our results obtained within a relatively short period of one year (during which nerve size rapidly decreased with resolution of CTS symptoms after reduction in serum IGF-I levels), it is likely that the enlargement of the median nerve in acromegaly is a disease-related process rather than the result of a compressive neuropathy.

IGF-I also plays an important role in body composition changes in acromegaly. Anabolic effects of GH/IGF-1 on skeletal muscles include suppressing locally synthesized myostatin and stimulating protein synthesis [24]. GH/IGF-I lipolytic effects result in a reduction in adipose tissue and sodium retention properties, leading to an increase in total body water and extracellular water [24]. The acceleration effect of excess GH/IGF-I on anabolism results in an increase in lean body mass in untreated acromegaly. Lean body mass decreases after successful treatment of acromegaly [15, 19, 25, 26]. Our study shows that patients with active acromegaly had higher lean body mass than controls. There was no significant difference in BMI and fat body mass.

In acromegalic patients, at baseline, the IGF-I level correlated with lean body mass. A yearlong treatment of acromegaly led to decreased lean body mass and increased fat body mass without changes in BMI. The changes observed in body composition in our study are in line with what was confirmed in previous studies in acromegalic patients using various methods of examination [16, 19, 27]. We did not confirm a correlation between BMI, fat body mass, and IGF-I levels. Our results seem to reflect that the “gains in body fat mass” are matched by losses in lean tissue. Some studies suggest that the reduction in lean body mass reflects the loss of extracellular water (ECW) and intracellular fluids rather than a true reduction in lean body mass [28, 29]. Hansen et al. found that a 4-week administration of octreotide led to a significant reduction in ECW [29]. O'Sullivan et al. demonstrated that a 3-month administration of somatostatin (octreotide) resulted in a decrease in an ECW of 2 kg [30].

To our knowledge, this is the first prospective study in newly diagnosed acromegalic patients focusing on the relationship between acromegaly, CTS, body composition, and their changes in response to acromegaly treatment. Our most important finding is that the changes in lean body mass are the direct effect of the overproduction of IGF-I, and they can play an important role in pathogenesis of CTS in acromegaly. At baseline, we confirmed a positive correlation between lean body mass and the median nerve CSA in acromegalic patients. The treatment of acromegaly led not only to a decrease in IGF-I levels but also to a decrease in lean body mass and to a significant reduction in CSA of the median nerve. Our finding of rapid reduction in the median nerve CSA at the 1-year follow-up after the initial acromegaly treatment (a relatively short period), as well as the decrease in lean body mass, supports the hypothesis that nerve swelling, rather than the increased volume of carpal tunnel contents, can be the cause of CTS in acromegaly.

In conclusion, we confirmed an enlarged median nerve in newly diagnosed acromegaly patients in comparison to healthy controls. Treatment of acromegaly at the 1-year follow-up led to a significant reduction in the median nerve CSA. These changes were closely associated with reduced IGF-I levels as well as lean body mass. Therefore, early and adequate treatment of acromegaly can improve median neuropathy and potentially avoid surgical treatment of CTS.

## Figures and Tables

**Table 1:** Baseline characteristics of subjects.

Characteristics	Acromegalic patients (*n* = 30)	Control group (*n* = 30)	*p* value
Sex (M/F)	12/18	12/18	
Age (year)	52 [43–59]	53 [42–61]	ns
GH (ng/ml)	6.50 [3.03–10.79]	0.19 [0.13–0.25]	<0.001
IGF-1 (ng/ml)	526 [372–686]	139 [113–162]	<0.001
Anthropometric measurements			
Height (cm)	171 [166–180]	168 [164–175]	Ns
Weight (kg)	89 [74–108]	89 [70–102]	Ns
BMI (kg/m^2^)	30.8 [25.0–34.1]	31.3 [25.3–34.8]	Ns

Ultrasonography			
CSA of the median nerve (mm^2^)	13.1 [12.2–14.9]	7.5 [6.4–8.4]	<0.001

DXA measurements			
Fat mass (kg)	30.1 [23.1–36.9]	31.3 [26.3–37.1]	Ns
Lean mass (kg)	60.4 [47.1–74.9]	49.1 [42.8–66.1]	0.033

Data are presented as median (standard interquartile range). The level of significance was set at ^*∗*^*p* ≤ 0.05. GH: growth hormone, IGF-1: insulin-like-growth factor 1, BMI: body mass index, CSA: cross-sectional area, and DXA: dual-energy X-ray absorptiometry.

**Table 2:** Comparison of biochemical characteristics, CSA of the median nerve, and body composition parameters in acromegalic patients at baseline and 1 year after treatment of acromegaly.

Characteristics	At diagnosis	1 year follow-up	*p* value
GH (ng/ml)	6.50 [3.03–10.79]	0.55 [0.34–1.29]	<0.001
IGF-1 (ng/ml)	526 [372–686]	210 [169–263]	<0.001
BMI (kg/m^2^)	30.8 [25.0–34.1]	30.2 [26.2–34.5]	Ns
CSA of the median nerve(mm^2^)	13.1 [12.2–14.9]	11.6 [10.2–13.1]	<0.001
Fat mass (kg)	30.1 [23.1–36.9]	30.7 [26.9–39.4]	0.038
Lean mass (kg)	60.4 [47.1–74.9]	60.9 [47.1–73.2]	0.032

Data are presented as median (standard interquartile range). The level of significance was set at ^*∗*^*p* ≤ 0.05. GH: growth hormone, IGF-1: insulin-like-growth factor 1, BMI: body mass index, and CSA: cross-sectional area.

## Data Availability

The data used to support the findings of this study are included within the article.
